# Prediction of the Maximum Temperature for Life Based on the Stability of Metabolites to Decomposition in Water

**DOI:** 10.3390/life5021054

**Published:** 2015-03-26

**Authors:** William Bains, Yao Xiao, Changyong Yu

**Affiliations:** 1 Earth, Atmospheric and Planetary Sciences, Massachusetts Institute of Technology, 77 Mass. Avenue, Cambridge, MA 02139, USA; 2 Department of Chemical Engineering and Biotechnology, University of Cambridge, Tennis Court Road, Cambridge CB2 1QT, UK; E-Mails: xiaoyao_ronnie@163.com (Y.X.); cam.ycy@gmail.com (C.Y.)

**Keywords:** thermophile, hyperthermophile, hydrolysis, decomposition, kinetics, metabolite, maximum temperature

## Abstract

The components of life must survive in a cell long enough to perform their function in that cell. Because the rate of attack by water increases with temperature, we can, in principle, predict a maximum temperature above which an active terrestrial metabolism cannot function by analysis of the decomposition rates of the components of life, and comparison of those rates with the metabolites’ minimum metabolic half-lives. The present study is a first step in this direction, providing an analytical framework and method, and analyzing the stability of 63 small molecule metabolites based on literature data. Assuming that attack by water follows a first order rate equation, we extracted decomposition rate constants from literature data and estimated their statistical reliability. The resulting rate equations were then used to give a measure of confidence in the half-life of the metabolite concerned at different temperatures. There is little reliable data on metabolite decomposition or hydrolysis rates in the literature, the data is mostly confined to a small number of classes of chemicals, and the data available are sometimes mutually contradictory because of varying reaction conditions. However, a preliminary analysis suggests that terrestrial biochemistry is limited to environments below ~150–180 °C. We comment briefly on why pressure is likely to have a small effect on this.

## 1. Introduction

Temperature is a physical state variable that has profound effects on all of chemistry, and hence on all of biology [1]. The limiting temperature above which life cannot flourish is of theoretical and practical importance to many biological and geochemical studies [2]. Terrestrial organisms are found to grow in a relatively narrow range of temperatures, between −20 °C and ~120 °C [1]. The lowest temperature compatible with life is set by the physical properties of water [3]. The upper limit is determined by the stability of the components of life.

We know that life can grow at temperatures of at least 120 °C (394 K) [4,5], and many microorganisms are known that grow between 100 °C and 110 °C. Some microbiologists have speculated that life could exist up to 140 °C [6,7,8], and *in situ* characterization of organisms in hydrothermal vent hints at active life up to 150 °C [9]. Organisms growing at extreme temperatures have similar biochemistry to mesophilic organisms such as ourselves, based on proteins, nucleic acids, carbohydrates, *etc*., and with much of their metabolic machinery functionally identical (reviewed in [10]). So adaptation to high temperatures involves many, relatively minor changes in biochemistry.

This paper addresses the limits of adaptation of terrestrial biochemistry to life at high temperature. We take an approach based on chemical structure. Chemical structures—molecules—are unstable as a result either of thermal cleavage of the bonds in the chemical or reaction of that chemical with others in its environment. The primary (bonding) structure of most small molecule biochemicals is chemically stable, so their instability at high temperatures is the result of reaction with other chemicals. By far the most abundant single chemical species in the living cells is water, and so attack by water, leading to hydrolysis or to other, water-catalysed reactions such as decarboxylation, is a major cause of instability of metabolites. The stability of organic chemicals to attack by water is a well-established field (reviewed in [11,12,13,14]) and we do not intend to review it here.

The structural approach has been discussed elsewhere [15], but usually in general terms either of the known stability of the biological macromolecules (see e.g., [7,16,17,18,19,20]) or of general statements on the stability of organic chemicals (see e.g., [21]). In this paper we extend both arguments by considering the stability of specific metabolites reported in the literature, and how that stability maps onto the requirements for stability of life.

This approach has precedent in the literature on thermophilic biochemistry. A number of studies have reported the stability of specific metabolites, often at a specific temperature (e.g., [17,22,23,24,25]). These often come to quite different conclusions about what the maximum temperature compatible with life might be. The disagreement is attributable to: (i) only using a few compounds that the author(s) thinks are relevant; and (ii) only using a few data points on stability, and not taking the uncertainties in that data into account. This paper provides a method for reconciling different studies, and for providing a measure of our confidence that the data available can be extrapolated. Our long-term goal is to extend the logic of this approach to include a representative selection of all of metabolism, to include experimental uncertainties in our projections, and hence to see how much of metabolism has to change to allow it to function at high temperature. This paper is the first step on that programme.

The current analysis is of *free metabolites* only. Amino acids, sugars and nucleic acid bases and their derivatives are also present in polymers that have critical biological function. We do not address the complex question of the stability of those polymers in this paper, but note that, no matter how stable an amino acid or a sugar is when it is incorporated into a protein or a polysaccharide, if it breaks down before it can be incorporated into the polymer then it cannot perform its function. So the kinetics of the decomposition of isolated metabolites is of relevance to all of biochemistry.

We describe our argument below as follows. After describing the methods used, we work through the example of the decomposition of two monosaccharide sugars in detail, to show the approach. We then apply the same approach to the decomposition of other sugars, amino acids, nucleic acid bases, and some other metabolites. We then summarise the results and produce an estimate of the likely maximum temperature for the stability of metabolism, and comment on the effects of pressure on that temperature. Lastly we discuss the limitations of this approach, and what further work is needed to make it more robust.

## 2. Method

Our approach uses literature data on the rate of reaction of metabolites or metabolite-like chemicals in water as the base for analysis. No new experimental data were generated for this paper.

### 2.1. Literature Information

Literature information about the rate of reaction was gathered through literature search. Two approaches were used. In the first approach, papers were identified by keyword search in Google Scholar^®^, including *hydrolysis*, *stability*, *subcritical*, *hydrothermal, kinetics* or combinations of them, and a list both specific chemicals names such as *glucose* and general terms such as *amino acid* or *metabolite*. The second approach was to identify all papers cited in the papers found by searching, and all papers that cited them. ~400 potentially relevant papers were identified this way. From these, data were extracted from papers that provided rate of reaction data for a metabolite in water or dilute buffer solution at neutral pH, which met specific criteria of data quantity and quality.

### 2.2. Exclusion Criteria

Our goal is to provide a quantitative measure of our confidence in the expected mean of a measured set of reaction rates at any temperature. To achieve this, several additional filters were applied to the papers selected for analysis here. To provide confidence limits on predictions of the reaction rate constant *k* as a function of temperature from one set of experimental data, at least three measurements of *k* at different temperatures are needed. With one exception noted below, only papers that provide measures of *k* at three or more temperatures were included. Only papers that followed a reaction over time to derive a reaction rate were considered: papers that took only one time-point and inferred a reaction rate from that were discarded. Papers that only reported Arrhenius kinetic parameters and not the reaction rate constants from which they were calculated also could not be used.

**Figure 1:**
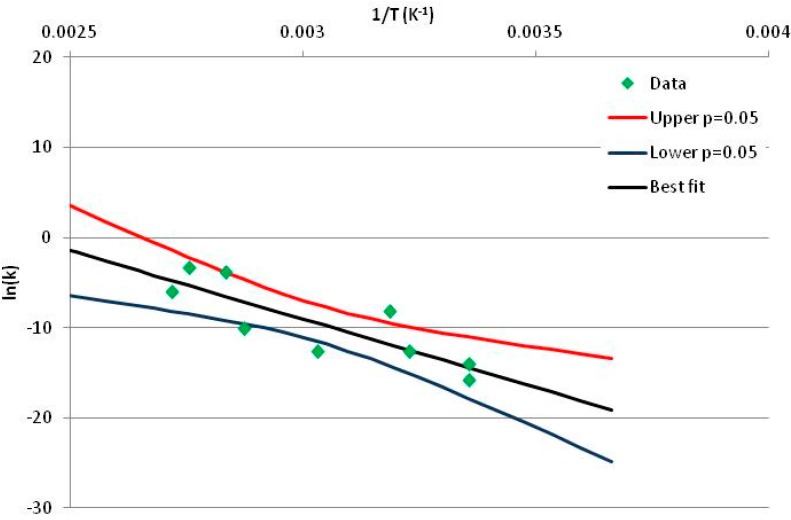
Plot of ln(*k*) *vs*. 1/*T* for the decomposition of NADH in phosphate buffer at neutral pH, from data reported in [27,28,29,30,31]. Black line—straight line of best fit. Red and blue lines—95% confidence limits on predictions of *k*, calculated as described in the Methods section.

The one exception to our criteria, and the illustration of their value, is the decomposition of reduced Nicotinamide Adenine Diclucleotide (NADH). Because NADH is a cofactor for many enzymes, its stability has been studied in at least five papers that are principally devoted to the study of enzyme kinetics, but also report data on NADH stability at one or two temperatures. (Some other papers report that they have examined NADH stability, but provide no data at all, e.g., [26]). Buffer ions and pH make a substantial difference to NADH breakdown [27,28], but the data at neutral pH in phosphate buffer can be combined to provide a consensus plot, which is shown in Figure 1. Figure 1 shows the substantial discrepancies between individually reported *k* values, but also shows that with a sufficiently large number of data points a linear relationship between ln*(k)* and *1/T* can be inferred, albeit with substantial uncertainty (nearly a factor of 30,000 in *k* at 130 °C). However, NADH is unique because of a uniquely large number of studies of its stability. For all other metabolites, each paper had to provide at least three temperature measurements, or be excluded.

Only papers that examined the decomposition of chemicals in neutral water (or neutral buffer) were considered. Acid- and base-catalyzed decomposition mechanisms are well known, and become increasingly important with increasing temperature, as the concentration of both H^+^ and OH^−^ ions in neutral water at neutral pH increases with temperature. We comment on the potential importance of this in the discussion.

Using these selection criteria, 52 papers were identified, and form the basis of this analysis.

### 2.3. Kinetic Analysis

The reaction of metabolites with water was assumed to be first order, because the concentration of water greatly exceeds the concentration of any other molecule in a living organisms (comprising 60%–80% of the organism by mass [32,33,34,35,36,37,38]), and so in a reaction between water and a solute the concentration of water is effectively constant. Thus

(1) $$rate=\;-\frac{d.\left[M\right]}{d.t}=k.\left[M\right]$$

and hence

(2) $$k=\frac{\ln\left(2\right)}{t_{½}}$$

where *rate* is the rate of reaction, *[M]* is the concentration of the metabolite under investigation, *t* is reaction time, *t_½_* is the half-time of the reaction, and *k* is the first order rate constant in dilute solution. The rate constant *k* is assumed to be related to the temperature of reaction by the Arrhenius equation

(3) $$k=A.e^{\frac{E_{a}}{R.T}}$$

and hence

(4) $$\ln\left(k\right)\;=\frac{Ea}{RT}+\ln\;(A)$$

where *A* is a constant (which includes consideration of the concentration of water in the reaction), *Ea* is the activation energy of the reaction, *R* is the Gas Constant and *T* is the absolute temperature. Where it was available, primary data was replotted to derive values for *k*, using Equation (2).

Ordinary linear least squares fitting provided gradient and intercept values for a line of best fit between ln*(k)* and $\frac{1}{T}$ as follows:

(5) $$\ln\left(k\right)=b.\frac{1}{T}+a$$

where *b* and *a* are constants (the gradient and intercept of the straight line respectively), and hence values for *A* and *Ea* can be calculated. The same data also provide confidence limits on the line of best fit, according to:

(6) $$boundary\left(\ln\left(k\right)\right)=\left[b.\frac{1}{T}+a\right]\pm \left[t.\sqrt{\frac{RSS}{n-2}}.\sqrt{1/\left(n+\frac{\frac{1}{T}-m}{\sigma_{T}}\right)}\right]$$

where *RSS* is the residual sum of the squares (*i.e.*, the sum of the squares of the difference between the actual data values and the values predicted by Equation (5)), *n* is the number of data points, *m* is the mean of the 1/T values, *σ_T_* is the variance in 1/T, and *t* is the t value for a two-tailed t-test distribution with n-2 degrees of freedom that has a probability of ≤p (calculated in Excel by t = TINV(p,n-2)). (From this, it is clear that confidence limits can only be estimated if we have at least three data points to analyze, hence the restriction on papers selected above). Confidence limits are calculated directly for a range of temperatures from Equation (6). We also explored the use of weighted least squares fit to the kinetic data, which takes the errors in estimating k into account: in general, this did not lead to systematically improved confidence [39,40], and so simple linear best fit was adopted as standard for this study.

The confidence limits so calculated are used in this paper as a measure of our confidence in estimating a mean rate from the data available. Specifically, they are the confidence that the mean rate found in a large number of thermal decomposition experiments will fall within a specific range. The method of calculation assumes that the experimental data is sampled from a single distribution of measured rates, and that the variability in the population of rates is Gaussian. When analyzing data from different experiments, these assumptions are likely to be false, and hence the confidence limits may under- or over-estimate our confidence bounds. If the assumptions are substantially at odds with the facts (as with the example of Xylose below) then the calculations will give inconsistent results. Thus we take the view that the confidence bands reflect a summary of our knowledge of a super-population of possible data sets from which it is assumed that these specific experimental data sets are derived.

The Stability Confidence Plots (*i.e.*, plots of our confidence that the reaction rate is no more than a specific target rate—see below) are calculated by iterating through all temperatures from 300 K to 640 K, for each temperature iterating through probabilities from *p* = 0.005 to *p* = 1, and finding the T and p values at which the upper or lower confidence limits most exactly match the target reaction rate. Note that this can involve extrapolating beyond the range of the measured data. The confidence limit calculations above can identify when this procedure is unreliable, assuming no systematic error in the data. If there are temperature-specific systematic errors in the data, then this extrapolation will be unreliable. In particular, if different decomposition mechanisms dominate at different temperatures, then the extrapolation will be unreliable.

All calculations were implemented in *Excel* spreadsheets.

### 2.4. Limits of Temperature Explored

All forms of biological matter are rapidly reduced to low molecular weight volatiles and a black, carbon-rich residue (“char”) by heating in water near water’s critical temperature of 647.1 K (373.9 °C) [41,42,43,44,45]. We therefore consider it not worthwhile to explore the reactivity of chemicals to water above 640 K (365 °C). Organic solutions such as bacterial and cell culture media and liquid-form pharmaceuticals can be stored without loss of complex biochemicals for months or years at room temperature if they are sterile, so we also do not consider it worthwhile exploring reaction rates below ~300 K (~25 °C).

## 3. Results

### 3.1. Illustration of the Method: Fructose and Xylose

We present analysis of the decomposition of the monosaccharide sugars fructose and xylose in some detail, to illustrate the approach, its power and its limitations.

**Figure 2:**
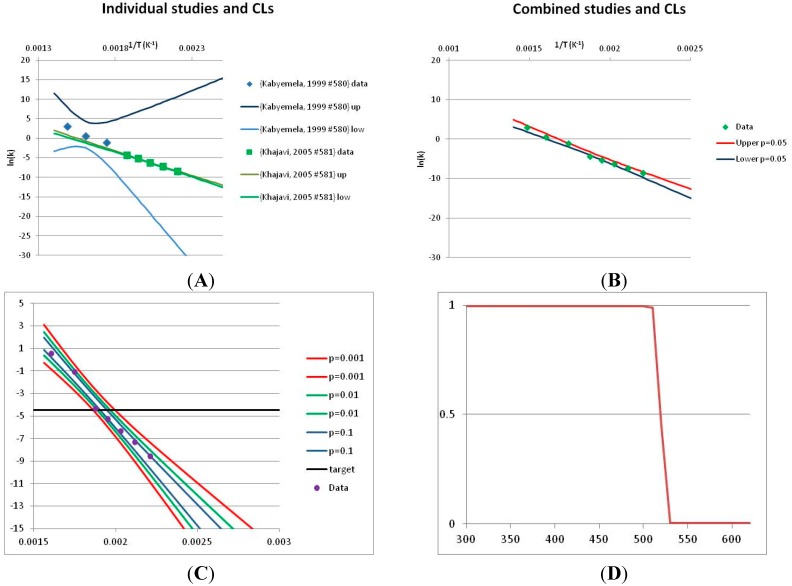
Hydrolytic decomposition of fructose. (**A**) Analysis of two papers, and individual 95% confidence limits in ln(*k*) calculated from the data for each paper separately. Data: actual measured data values for ln(*k*) *vs*. 1/T. “up”—upper 95% confidence limit. “low”—lower 95% limit; (**B**) Analysis of data from two papers combined, and 95% confidence limits on the value of ln(*k*) derived from the combined data; (**C**) Plot of combined fructose decomposition data vs. ln(*k*) values, with different confidence limits; (**D**) Stability confidence plot for fructose for a 60 s half-life.

The analysis of the decomposition of fructose is illustrated in Figure 2. Two papers were found with appropriate kinetics data for the decomposition of Fructose [46,47]. *Kabyelmela et al.*, measured reaction rates at three temperatures, *Khajavi et al.*, at five temperatures. The *k* values at different temperatures and the 95% confidence limits on predicting a kinetic constant *k* from those data points are shown in Figure 2A. Because there are only three temperatures analyzed in [46], the confidence limits on *k* calculations for this data set are wide even within the range of temperatures sampled in the experiment, and reaction rates cannot be usefully extrapolated beyond the experimentally sampled range. The data from [47] show a much narrower 95% confidence range, but the data from [46] fall outside that range, suggesting some inconsistency between the two studies.

The data from the two experiments can be pooled, and analyzed as a single data set. We note that the interpretation of the Confidence Intervals in this pooled analysis assumes that the experiments were exact replicates (*i.e.*, the data points can be taken as being from the same experiment), and clearly this is not the case. However, we believe that the Confidence Interval calculation nevertheless provides a guide to the consistency of the data which: (a) can be used consistently across studies; and (b) is capable of quantitative comparison. Pooling the data is performed in Figure 2B. Even though the two experiments are not completely consistent, the confidence limits in predicting *k* from the pooled data set are much narrower than from either individual set, because of the larger number of data points analyzed. We note that this is a slightly surprising result, but is an accurate reflection of our *confidence* in the trends found in the data. The confidence limits reported here could be an over-estimate of the spread of data (because our confidence is reduced by un-identified systematic errors in one data set), or an under-estimate (because of biases common to more than one experiment). A specific bias might be that in the combined data set the left-most and right-most points have considerable influence on the outcome, and may disproportionately affect the calculation. We cannot distinguish between over- and under-confidence possibilities from the published data as analysed here, so here we use the confidence limits as simple measures of our confidence in the consistency of the data, and hence our confidence that we can interpolate within or extrapolate from that data. This is the meaning of confidence limits in this context—our confidence that, *on the assumption* that this data is an unbiased, random sample from an infinite set of measurements of decomposition rates, we can interpolate and extrapolate an expected average decomposition rate for any given temperature. On this basis, we would be confident in using this extrapolation to predict a reaction rate outside the range sampled by the experimental data. We discuss the limitation of this interpretation further in the Discussion section below.

We can use the analysis shown in Figure 2B to give a measure of confidence that fructose is stable at a given temperature. To perform this calculation, we need a measure of what ‘stable’ means: to illustrate the method, we will take “stable” to mean “having a half-life of hydrolytic destruction of at least 60 s”, *i.e.*, from Equation (2),

(7) $$k\leq \frac{\ln\left(2\right)}{60}=0.01155,\;\ln\left(k\right)\leq -4.46$$

We can replot Figure 2B to show a number of confidence bands, and see where the k = −4.46 line intersects each confidence level. (We note that this value lies within the range of temperatures studied, and so does not require extrapolation in this case.) This is done in Figure 2C. By reading across the figure along the line of ln(*k*) = −4.46, we can see how confident we are that the mean of the ln(*k*) calculated from a large number of experimental data sets actually will be less than −4.46 where the horizontal line intercepts each confidence band. Such an analysis allows us to determine the confidence we have that k will be found to be *no more than* −4.46 at a given temperature. We can invert this analysis, and plot our confidence that ln(*k*) is no greater than −4.46 as a function of temperature. This last graph is plotted in Figure 2D. Figure 2D shows that we can confidently predict that a large number of measurements of the stability at different temperatures will show that Fructose has a half-life of at least 60 s at ~520 K (~240 °C, 1/T = 0.0019) or below. We term the type of plot shown in Figure 2D—a plot of confidence in prediction of a threshold rate constant *k* as a function of temperature at which *k* is predicted—a Stability Confidence Plot (we note that this implies a confidence in stability rather than in the mean measured stability rates, but for conciseness we will use this shorter, less precise terminology hereafter). Because the data on fructose is broadly consistent between the two studies, we are confident that we can estimate a temperature at which fructose will have a half-life of 60 s or more within a narrow band of temperatures.

**Figure 3:**
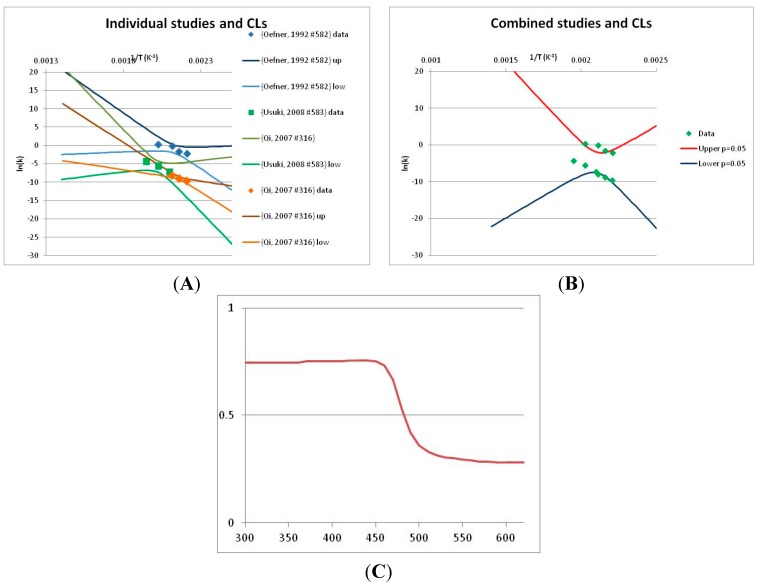
Decomposition of Xylose. (**A**) Analysis of three papers, and individual 95% confidence limits (CL) in ln(*k*) calculated from the data for each paper separately. Data: actual measured data values for ln(*k*) *vs*. 1/T. “up”—upper 95% confidence limit. “low”—lower 95% limit; (**B**) Analysis of data from three papers on xylose decomposition combined, and 95% confidence limits on the value of ln(*k*) derived from the combined data; (**C**) Stability Confidence Plot for xylose for a 60 s half-life.

The analysis of xylose is illustrated in Figure 3. Three papers provide relevant data. Ref. [48] provides four temperature points, refs. [49,50] each provide three. The primary data and confidence limits on ln(*k*) predictions are shown in Figure 3A, and the combined data set is analyzed in Figure 3B. Because [48] gives a very different intercept of the line of ln(*k*) vs. 1/T to that of [49] and [51], combining the data reduces our prediction confidence. As a result, our Stability Confidence Plot (Figure 3C) shows we have limited confidence in a prediction of the stability of xylose at any temperature. We note again that this is a measure of our confidence that, if we assume the individual data points are representative of all decomposition rates, we can predict the average decomposition rate at different temperatures.

It is noticeable that the data from references [49,50], from two different groups, follow a coherent pattern in Figure 3A, while the data from reference [48] follows a different pattern. Are we justified in discarding one or other of these data sets, and so producing a consistent set of data? There are no major differences between the conditions of the experiments: all were done in water at roughly neutral pH in a steel pressure vessel. Study [48] used substantially older analytical technology, and their data are noisier (as witnessed by the wider confidence limits in Figure 3A). So we might argue that we should discard the data from [48]. However [48] measures all the products of decomposition, whereas [49,50] measured only furfural production, so if furfural is a minor product, or is the end result of a complex reaction series, its production might be materially slower than the initial breakdown of xylose, and so we should use the [48] data alone. In the absence of a compelling reason for selecting one or other data set, we are forced to use all the available data (or none), and come to a correspondingly ambiguous conclusion.

We emphasize that our conclusion does not imply that any of the studies on xylose decomposition are poorly conducted or are incorrect. All three studies were carefully done and all are relevant for the purpose for which they were conducted. All this analysis shows is that there is insufficient data in the published literature to draw general conclusions about the stability of xylose that can be applied to the prediction of the maximum temperature for life.

### 3.2. Definition of Stability

To compare the predicted stability of cellular components with that needed for life, we need a definition of “stable”. “Stable” is a relative term. Snowballs are stable in a furnace if we consider a timescale of microseconds, diamonds are forever only if ‘forever’ is less that the order of ~10^880^ years [52,53]. Here we define “stable” as “having a half-life to thermal degradation greater than the average residence time a component of the cell needs to have to perform its function in the cell”. This is the target stability.

Identifying the target stability for a component of the cell is complex, due to five, interacting factors. The component must be available to the cell long enough to perform its function. This time is a function of the rates of synthesis and use of the component. The time is also related to the extent to which the component’s function can be replaced by another component so that a reduced level of the component can be tolerated. A third, and often neglected, factor is that the degradation products of the component must not build up to a sufficient degree to interfere with cellular metabolism, so the rate of operation of the mechanism to remove degradation products must at least equal the rate of the degradation product formation. (We note that this might be a trivial constraint, as is the case when a breakdown product is CO_2_, but can be a material constraint as in the formation of cyanate by the thermal breakdown of carbamoyl phosphate [54], or the formation of the toxic metabolite NADHX from NADH, which is sufficiently toxic to require specific enzymes for its removal and re-conversion of NADH in eukaryotes [55]). Fourthly, all of these factors change with cell genotype and growth rate, and so no general solution is possible. And lastly, many components have more than one function in a cell, and hence more than one target half-life.

To provide a first pass estimate of the maximum temperature for life, we chose the metabolic pool turnover time for each metabolite in exponentially growing *E.coli* cells as a target half-life. There are few metabolites for which thermal stability data is available and for which metabolic pool turnover rates are known, and so we have grouped metabolites into functional classes and assigned an average pool turnover to each class. (We group them into functional classes because it is plausible that the metabolites in one interconnected metabolic pathway will have similar turnover times to others in the same pathway.) Table 1 summarizes the pools used, and lists two target half-lives for each pool, representing high (long half-life) and low (short half-life) values derived from the literature. By comparing the results obtained from both values, we can show that our conclusions are not strongly sensitive to choice of target half-life. More detailed information on individual metabolite pool turnover times is provided in the Appendix. We appreciate that this is a very crude analysis of the stability criteria for metabolites. However, as we show below, the exact numerical value for the target metabolite turnover time does not have a substantial effect on our conclusions.

**Table 1:** Summary analysis of reports on stability of metabolites in water.

Category	Code	Specific Metabolites Analysed in This Study	Half-Life		References for Half-Life
			Long	Short	
Amino acids	A	Alanine, Alpha-amino butyrate, Asparagine, Aspartate, Glycine, Histidine, Isoleucine, Leucine, Methionine, Phenylalanine, Proline, Serine, Threonine, Tyrosine, Valine	200	20	[56,57,58,59]
Sugars	S	Arabinose, Cellulobiose, Fructose, Galactose, Glucose, Isomaltose, Lactose, Lyxose, Maltose, Mannose, Melibiose, Palatinose, Ribose, Sucrose, Trehalose, Turanose, Xylose	600	60	[60]
Sugar derivatives	D	Glucosamine, N-acetyl glucosamine,	(a)	(a)	
Lipids	L	Long chain triglycerides, Phosphatidyl choline	500	5000	[61,62]
Nucleotides and their components	N	Adenine, Adenosine, Adenosine, Cytidine, Cytosine, Deoxyadenosine, Guanine, Thymidine, Uracil	100	10	[15]
Glycolytic intermediates	G	Dihydroxyacetone, Glyceraldehyde, Fructose-1,6-diphosphate, Pyruvate	10	1	[63,64,65]
Tricarboxylic acid cycle intermediates	T	Fumarate, Oxaloacetate	10	1	[60,63]
Other intermediary metabolism components	I	2-Dimethylaminoethanethiol Propionate (thioester analogue), Carbamoyl phosphate, Formic acid, Hypoxanthine, Malonate, Mandelic Acid, Orotic acid, Urea, Xanthine	(b)	(b)	
Energy carriers	E	AMP, ATP	30	3	[60,66]
Other carrier molecules	C	Coenzyme A, NADH	500 (c)	50 (c)	[67]

Note that the references are for background on why these are plausible values to select. They are not references to a consistent set of experiments that exactly validate these values as “correct”. The code in column 2 is used in the Appendix to identify which class of compounds a metabolite falls into. Notes. (a): No data found, assumed to be the same as sugars. (b) No data found, assumed to be the same as Glycolytic and TCA intermediates. (c) The turnover of NADH referred to is the synthesis of NAD^+^/NADH, not their interconversion, as heating will destroy the core nicotinamide ring rather than reduce or oxidise it [28].

### 3.3. Summary of Results

The reader will probably be grateful that we have not elaborated the analysis of the other data explored in this study as fully as the analysis of fructose and xylose above. Table 1 summarizes the compounds analyzed in this paper. The Appendix provides the data plots from all these compounds, the per-study confidence limits, Stability Confidence Plots of aggregated data where more than one study was analyzed, and the temperatures at which the Stability Confidence is 0.5 for each of the long and short half-life cases.

**Figure 4:**
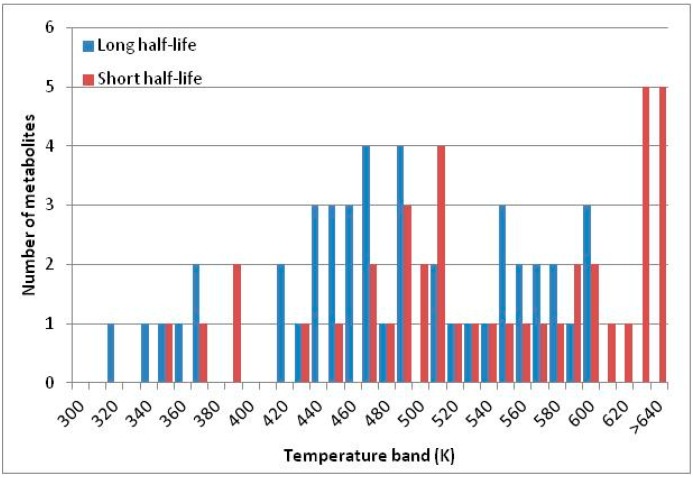
Stability of metabolites as a function of temperature. X axis: temperature at which there is a 50% chance that the means from a large number of experiments measuring decomposition rates would be no more than a longer target half-life (blue bars) or a shorter target half-life (red bars), binned in 10 degree bins. The number on the X axis is the lower end of the bin (*i.e.*, “300” means a bin of 300–309.9 K). Y axis: number of metabolites showing that stability.

**Figure 5:**
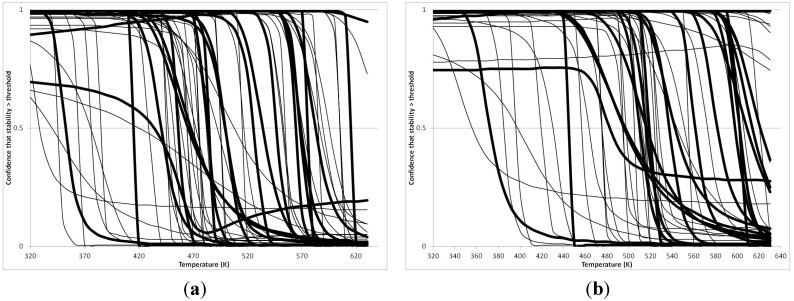
Stability Confidence plots for all metabolites analysed. Y axis: Confidence that the means of half-lives in repeated thermal decomposition experiments would be expected to be at least the target half-life listed in Table 1. X axis—temperature (K). Bold lines are for metabolites for which two or more papers have been analysed. Thin lines are for metabolites for which only a single paper’s data could be identified. (**A**) Long half-life (low temperature) threshold; (**B**) short half-life (high temperature) threshold.

Figure 4 summarizes the temperatures at which the metabolites analyzed in this paper have a 50% chance of being stable at the long- and short-half-lives listed in Table 1. Figure 5 shows the Stability Confidence Plots for all the metabolites analyzed, at both target half-lives. The identity of the metabolites with less than a 95% confidence of stability at 420K (147 °C) is shown in Table 2. Table 2 shows several metabolites that are known to be unstable at high temperatures (NADH, Carbamoyl phosphate, ATP), but also lists a number of surprising molecules such as sucrose and triglycerides. We discuss the implications of these in the section below.

**Table 2:** Low stability metabolites

Metabolite	Short Half-Life Vulnerable?	Category
2-Dimethylaminoethanethiol Propionate (thio-ester analogue)	√	Labile
Asparagine	-	Labile
ATP	-	Labile
Carbamoyl phosphate	√	Labile
Deoxyadenosine	-	Labile
Glyceraldehyle	√	Uncertain
Histidine	-	Uncertain
Long-chain triglycerides	√	Uncertain
Lyxose	√	Uncertain
NADH	√	Labile
Oxaloacetate	√	Uncertain
Palatinose	√	Uncertain
Sucrose	√	Uncertain
Xylose	√	Uncertain

Metabolites with a <95% confidence that the mean decomposition rate from repeated experiments would be less than their long half-life stability at 420 K (147 °C). Column 1: metabolite. Column 2: √ indicates that we are less than 95% confident that the mean decomposition rate from repeated experiments for this metabolite would be greater than a short half-life target. Column 3: why the metabolite fails to achieve 95%. “Lability”—Stability Confidence plot is steep, failure to achieve 95% confidence at 400K is probably due to genuine lability of the molecule. “Uncertain”—Confidence plot is shallow or does not stretch from 0 to 1, and failure to achieve 95% confidence is probably due to inadequate data, and may not reflect lability of the molecule.

## 4. Discussion

In this paper we describe an approach to providing a quantitative estimate of our confidence that specific metabolites can be stable enough to allow life to persist at any temperature. Specifically, we provide a confidence that a large number of measured mean rates will not exceed a threshold value. Our approach integrates this confidence with the half-life that a metabolite has to achieve to be useful to life into an overall estimate of whether a metabolite is stable enough to be function in a cell.

The metabolites analyzed here are not a systematic set. Rather, they are all the metabolites for which data is available on their thermal stability in water. With this substantial caveat, we can draw some preliminary conclusions about the maximum temperature at which intermediary metabolism could be stable.

A simplistic reading of Figure 4 suggests that life is not possible above 100 °C, because at least two metabolites from this set are unstable above 373 K. Clearly life *is* possible above 100 °C. However, this contradiction does not invalidate our analysis.

Studies of reactions involving NADH and carbamoyl phosphate in hyperthermophiles show that prokaryotes that live at 110 °C or higher can adapt their metabolism to reduce the impact of the breakdown of these labile metabolites, by substrate channeling of highly labile intermediates [68,69,70] or by swapping labile metabolites for less labile ones [7,71,72,73,74]. The former reduces the time that a metabolite has to survive before it is used and protects it from attack by water in a closed, hydrophobic enzyme environment. The extensive binding of metabolites to proteins inside the cell may also protect them from water. The latter substitutes a labile chemical function (e.g., the redox carrying function of NADH) for a less labile molecule that can carry out the same function (e.g., an iron-sulfur protein) [71].

We would expect living organisms to adapt to any temperature by similar mechanisms, that is to say by exchanging the small fraction of their metabolic components that are labile at that temperature for other, chemically equivalent but more stable metabolites. The maximum temperature that the organism can tolerate then becomes the temperature at which the most labile component of its new metabolism ceases to have the required stability. It is reasonable to propose that there is a limit of such adaptation. In the example of replacement of NADH above for an iron-sulfur protein, to adapt to an even higher temperature the organism would have to exchange its iron-sulfur protein for an even more stable chemical (assuming the iron-sulfur protein was the most labile metabolite in the cell). Figure 4 and Figure 5 both show a cluster of metabolites with maximum stability temperatures around 420 K (high half-life) and 450–460 K (short half-life). Below these temperatures a small fraction of the set of metabolites is unstable, and we might plausibly suggest that they could be replaced by other, more stable metabolites. Above these temperatures the fraction of unstable metabolites rapidly rises to 50%, and it is implausible to suggest that metabolism could be adapted to replace them all with more stable molecules of equivalent function. This suggests, from Figure 4 and Figure 5 and Table 2, that the temperature at which intermediary metabolism may become unstable to water-mediated decomposition lies around 420 K (150 °C) if the long half-life target stabilities are taken as a target, 450–460 K (180 °C) if the short half-life stabilities are the appropriate reference.

An unexpected implication of our findings is that the lability of lipids may be a limiting factor on the stability of central metabolism, even though they are intrinsically much more stable than molecules such as ATP. This is because lipids have a much slower turnover than ATP, as they are structural components of the cell. Hyperthermophilic archaea use ether-linked lipids in place of ester-linked lipids, although ether-linked lipids are not unique to this clade. Our analysis here suggests that increased chemical stability of ether-linked lipids might indeed be a reason for their greater use in hyperthermophiles, as was originally suggested on their discovery [75], despite subsequent analysis that has called this into question [76].

### 4.1. Pressure Effects

Many of the kinetic measures analyzed here have been performed under high pressure. Pressure can change the rate of a reaction by stabilizing or destabilizing its transition state [77]. In general, solvolysis reactions are faster under high pressure because pressure reduces the solvation volume around transition state, and structures solvent around separated charges [78]. The change in the rate of reaction is related to the Activation Volume (ΔV^‡^, the difference between the volume to the reactants and the volume of the transition state, including any solvating molecules) [77,79]. Empirically, for hydrolysis reactions in water involving uncharged species, we find

(8) $$\ln\left(\frac{k_{l}}{k_{h}}\right)=-1.562.10^{-11}.{\left(\Delta P.\Delta V^{‡}\right)}^{2}-4.18.10^{-5}.\left(\Delta P.\Delta V^{‡}\right)$$

where *k_l_* is the reaction rate at low pressure, *k_h_* is the reaction rate at high pressure, ∆P is the change in pressure in atmospheres and ∆V^‡^ is in cm^3^/mol. (Modelled from data from [80,81,82,83,84,85,86,87,88,89,90]). ∆V^‡^ typically falls in the range ± 20 cm^3^/mol for organic chemical reactions [79,88], which implies that the change from atmospheric pressure to the critical pressure of water (217 atmospheres) will at most change the rate of a hydrolytic reaction by a factor of 20%, and change from the sea’s surface to the pressure at the bottom of the Challenger Deep (10,900 m, pressure 1100 atmospheres) would speed up hydrolysis ~2.3 times.

We can compare this with the change in rate caused by an increase in temperature. The “Q_10_”—the amount a reaction is increased by a 10 °C raise in temperature—is generally taken to be 2. In fact this specific number is an artefact of how early kinetic experiments were done [13], and is a function of temperature. The Q_10_ of the set of molecules analysed here averages 5.65 between 30 °C and 40 °C, 2.4 between 140 °C and 150 °C. Thus the effect of the highest hydrostatic pressures on Earth on the rate of hydrolysis of metabolites would be similar to a 10 °C increase in temperature at the likely stability limit of metabolism. This result is confirmed by experimentation. While high pressure is an effective sterilization mechanism [91,92,93,94], it works by destabilizing macromolecular complexes rather than breaking down their chemical components, which is why it is used for sterilizing food without changing the complex chemistry that leads to the food’s taste (reviewed in [95]).

We conclude that, within the bounds of the pressures that life is likely to experience on Earth, pressure will have a minor role on the chemical stability of metabolism compared to temperature.

### 4.2. Limitations of This Study

This study has several major limitations, which we lay out in detail below in the hope that others can overcome them. The most obvious is that we address only isolated metabolites in dilute solution, and neither consider metabolites in protein-rich environment of the inside of a cell nor consider the stability of the cell’s polymers. The stability of polymers, and of multi-molecular assemblies such as membranes, has been addressed elsewhere. Our goal here was to address small molecules only, but to do so systematically.

More practical but nevertheless still severe limitations derive from our dependence on literature data, *i.e.*, on the measurements that others have made, usually for purposes other than elucidating temperature effects on biochemistry, which leads to six issues.

Firstly, many metabolites have never been studied for their stability to sufficient depth to make statistically defensible predictions about their stability at a range of temperatures. A very intense search found only a few studies of phosphorylated metabolic intermediates. Many metabolites in intermediary metabolism are phosphorylated, but only seven phosphate-containing metabolites are included in the set analyzed in this paper. Key metabolic intermediates such as triose phosphates, Phospho-enol-pyruvate, citrate, α-keto-glutarate, acetyl-CoA, NADP^+^ and folate are not represented. There are also no authentic thioester metabolites listed here—the model compound 2-Dimethylaminoethanethiol propionate was included as a thioester metabolite analogue, but this is scarcely satisfactory. There is a substantial literature on the hydrolysis of thioester compounds, but the research focuses on variation of the rate of hydrolysis with pH, and those few that provide kinetic data at different temperatures do so at highly acid or alkaline conditions. Clearly, if we are to model the failure of metabolism with temperature, we must have a better data set than a handful of amino acids and sugars.

Secondly, of the compounds that have been reported in more than one paper, ~6/20 yield conflicting results (the exact number depends on the amount of conflict that can be accepted), for reasons that are not apparent from the methods sections of the papers reporting the studies. These compounds have broad, flat Stability Confidence Plots, sometimes ones which do not reach confidence above 0.9 or below 0.1. Such plots are a reflection of the inability of this method to derive a consistent prediction of the stability of the chemicals concerned. Other statistical methods may be more successful; exploring different statistical approaches to extracting more value from the literature data would be a valuable extension to this work.

Thirdly, we have not considered that different mechanisms or even different pathways of decomposition may dominate at different temperatures. For example, at high temperature (where neutral water has a pH < 7), acid-catalyzed mechanisms may dominate, whereas at cellular temperatures neutral water attack may dominate. Extrapolating from measurements at high temperature to lower ones may therefore not be valid. This is less of a concern than the other limitations, as measurements of decomposition for the more labile metabolites have been carried out in the 100–150 °C range, *i.e.*, the temperature regime in which we are interested. This again emphasizes that additional measurements, in this case at temperatures around those expected to be the maximum for life, would add confidence to the analysis done in this paper.

Table 2 illustrates the impact of this uncertainty. The right-hand column of Table 2 lists whether a molecule is classified as potentially unstable (<95% confidence of stability at 420 K) because it has a sharp, clearly defined decline in our confidence of prediction of the measured mean rate of decomposition with temperature below 420 K, or because it has a broad, shallow decline in confidence (*i.e.*, we have no confidence in a prediction). Eight out of 14 metabolites listed in Table 2 are there because their data is too uncertain to make a clear call on their stability at 420 K. We emphasize that this is a true reflection of the data: we might speculate based on a specific data point from one of the papers concerned that histidine or xylose is stable at 420 K, but this is not statistically supported by all the evidence available.

The only solution to all the caveats above is more, consistent data.

Fourthly, we have relied on studies of isolated molecules in dilute solution. This is not a very good model for the inside of the cell, which is packed with protein and with other metabolites. Molecular mobility and water activity within a cell are substantially different from that in free, dilute solution, and may dramatically stabilize some molecules. In addition, metabolites may actively bind to proteins, reducing their lability to attack by water. We have also selected decomposition rates at neutral pH: while neutral pH is a reasonable approximation of the inside of a cell, a more refined model would take intracellular pH into account explicitly.

Fifthly, hydrolysis and many other water-mediated decomposition reactions in dilute solution are effectively irreversible. In concentrated solution, however, or in solutions of low water activity, re-condensation of hydrolytic products can occur, and so compounds that hydrolyze readily in very dilute solution can be dynamically stable in more concentrated conditions [96,97]. This is a thermodynamic problem, not a kinetic one, but should also be addressed by a more realistic model of the interior of the cell.

Lastly, this study has not addressed the reaction of metabolites with each other. The reaction of amines with carbonyls is a well-known chemistry which happens on a timescale of hours during cooking at 100–110 °C, but is reduced in hyperthermophiles by several mechanisms, including removing target chemistry, substrate channeling and increased levels of scavenger molecules [98]. However, the literature on the kinetics of such reactions is even more limited than that on first order decomposition.

We note that the statistical methods used here, while appropriate for estimating confidence in single experiments, are being stretched beyond their rigorously valid application in being applied to data pooled from several papers unless we assume that each experimental data set is a sample from a super-population of possible data sets. More sophisticated statistical analysis would provide more robust confidence calculations, and we would welcome further work on this.

## 5. Conclusions

We have analyzed the literature data on the kinetics of water-mediated decomposition of 63 metabolites, using a model that takes experimental variability into account to give a confidence limit on whether a compound is stable at a given temperature. We have used literature data on decomposition rates, which has a number of substantial limitations. Despite these, we tentatively identify 150–180 °C as a maximum temperature for terrestrial life, based on the stability of its low molecular weight constituents. This study points up the need for an improved set of kinetic data to provide more robust predictions of stability of metabolites, and hence the thermal weak points in intermediary metabolism.

## Appendix

Table A. Data on individual metabolites analyzed in this study. Column 1: Metabolite. Note that all metabolites for which three-temperature decomposition kinetics were identified are listed in this appendix, but some did not yield usable kinetic data and so are not included in the analysis in the main text. These exceptions are noted in Column 6. Column 2: category of metabolite, used to choose a target half-life according to Table 1. Column 3. Reference(s) for kinetic data. Column 4: Plot of combined data and 95% confidence limits on prediction of ln(*k*) from that data. Column 5: notes. Column 6: metabolic half-life found for that metabolite (if any). Column 7: reference for metabolic half-life. Column 8: Stability confidence Curve for metabolite at the two target half-lives. Column 9: temperature at which Stability confidence = 50% for the longer half-life (*i.e.*, mid-point of blue curve in Column 8). Column 10: temperature at which Stability Confidence = 50% for the shorter half-life (*i.e.*, mid-point of the red curve in Column 8). Graphs in column 8 are plotted with the same X axis in each case for ease of comparison, which means that some of the more stable compounds predict curves that do not go to zero within the temperature range of the graph.

**Table A:** Summary data on metabolites analysed.

Compound	Metabolite Group Code	Degradation References	Combined Data	Degradation Notes	Metabolic Half-Life	Ref for Half-Life	Stability Confidence Curve	T(*p* = 0.5) – long	T(*p* = 0.5) – short
2-Dimethylaminoethanethiol Propionate (thio-ester analogue)	I	[99]		Included as an analogue of Thioesters				345.7	384.7
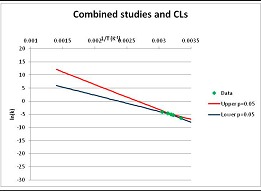
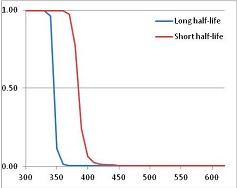
Adenine	N	[100]						586	668.8
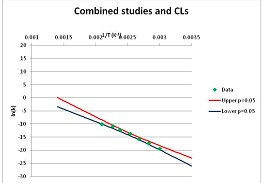
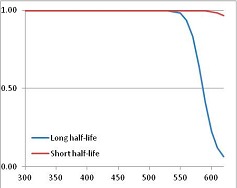
Adenosine	N	[101,102]		Includes deamination and cleavage of glycosidiv bond				482.9	513.9
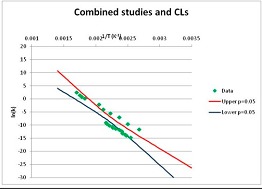
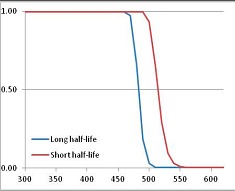
Alanine	A	[103,104,105,106]			20	[56]		568.4	597.8
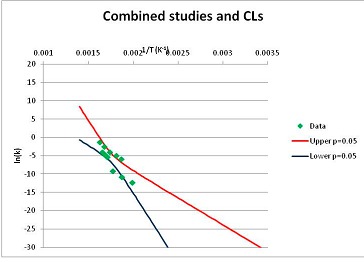
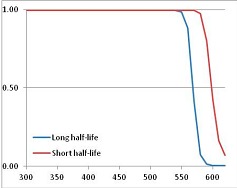
Alpha-amino butyrate	A	[105]						586.7	620
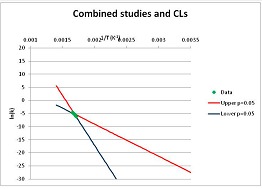
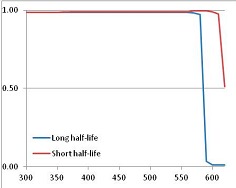
AMP	E	[101]			120	[60]		440.7	475.7
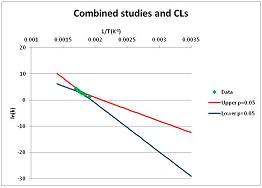
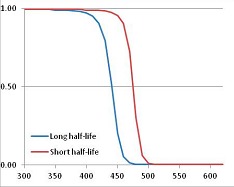
Arabinose	S	[49]		Marginal data quality, analysed anyway.				480.6	528.5
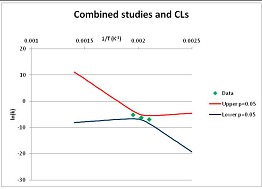
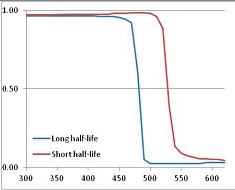
Asparagine (Side-chain)	A	[107]						385.4	419.8
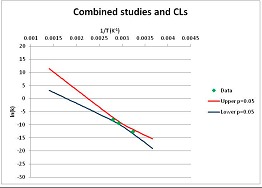
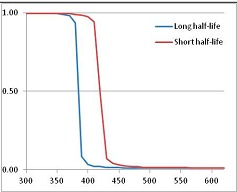
Aspartate	A	[106,108,109]			150	[56]		499.3	542.4
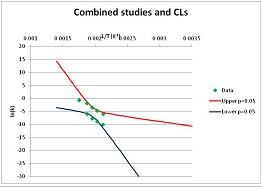
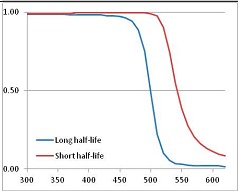
ATP	E	[25,101]			45 1	[60,66,110]		414.5	442.3
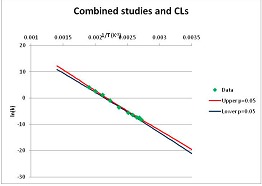
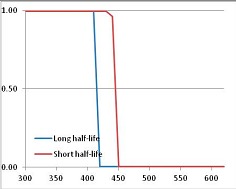
Carbamoyl phosphate	I	[55]			5			362	396.7
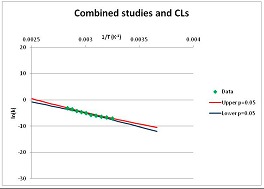
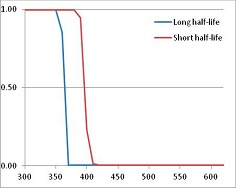
Cellulobiose	S	[111]						477.9	513.2
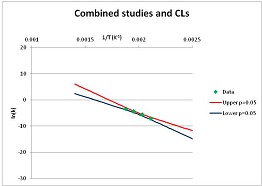
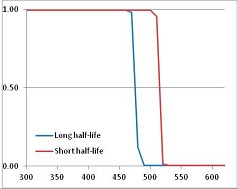
Coenzyme A	C	[112]				120		507.6	699
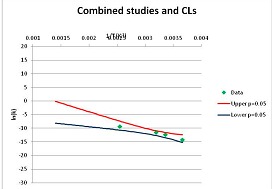
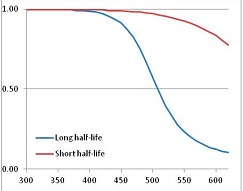
Cytidine	N	[113] [skipped – inconsistent with three others] [114,115,116]		Data from Snider was inconsistent with other data sets, and so was excluded				465.1	497
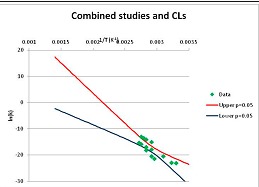
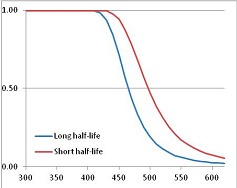
Cytosine	N	[100,114,117]						467.7	495.8
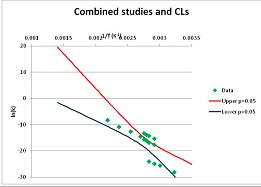
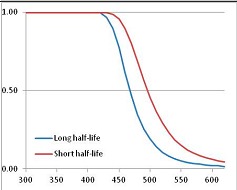
Deoxyadenosine (deamination)	N	[118]						377.5	463
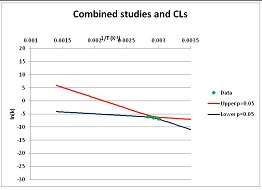
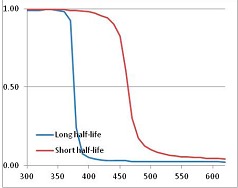
Dihydroxyacetone	G	[119]			271 2.7 10	[63] [64] [65]		552.5	654.8
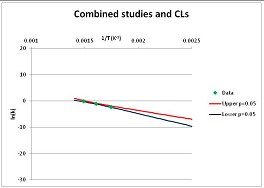
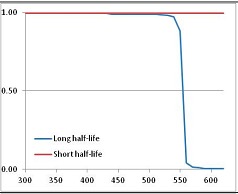
Formic acid	I	[120,121]						614.4	692.6
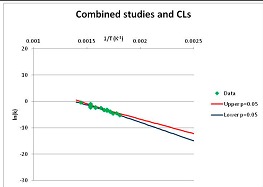
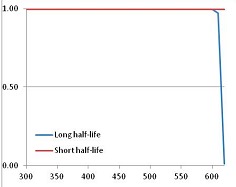
Fructose	S	[46,47]						484	519.6
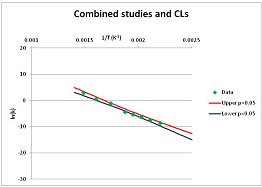
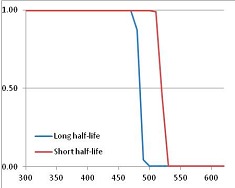
Fructose-1,6-diphosphate	G	[122]			0.6 10	[64] [65]		467.1	511.8
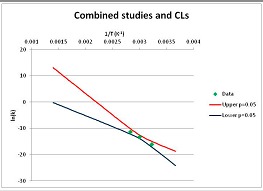
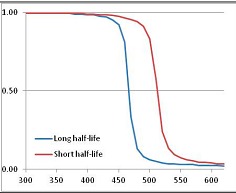
Fumarate	T	[123]			310 0.9 4	[63] [64] [124]		643	720.1
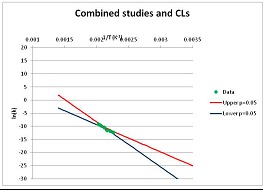
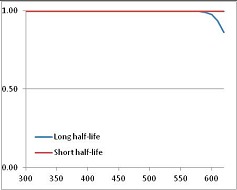
Galactose	S	[47]						495.6	520.6
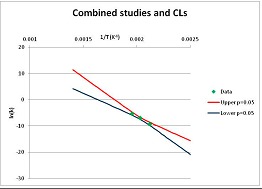
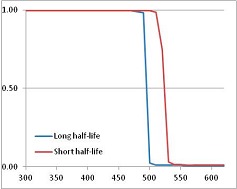
Glucosamine	D	[125]						492.1	551.3
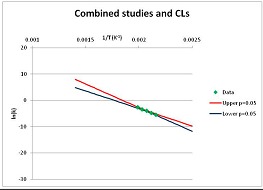
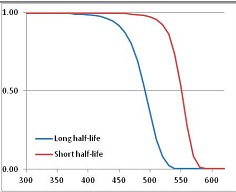
Glucose	S	[46,47,50]			600	[60]		499.6	537.3
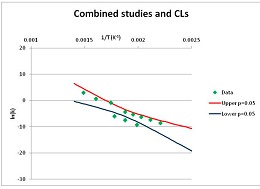
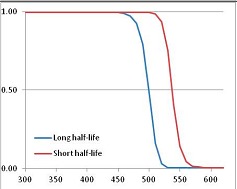
Glyceraldehyde	G	[119]			2.7 10	[64] [65]		420.9	685.3
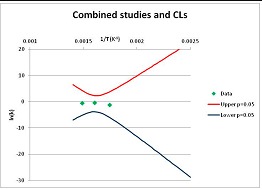
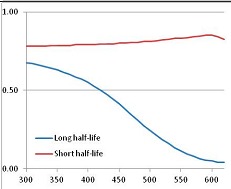
Glycine	A	[103,105,106,126,127]			30	[56]		533.9	608.8
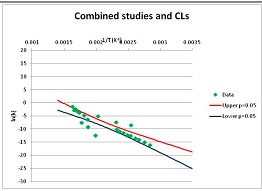
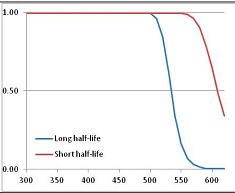
Guanine	N	[100]						551.5	605.7
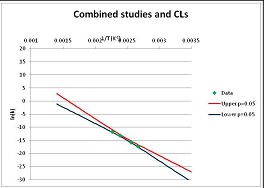
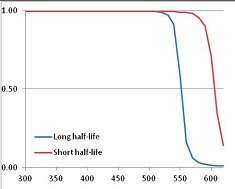
Histidine	A	[105,128]						873.6	7626.1
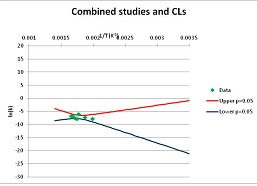
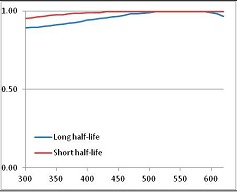
Hypoxanthine	I	[100]						516.9	572.2
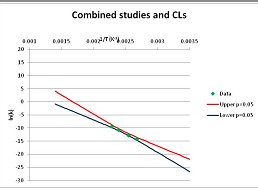
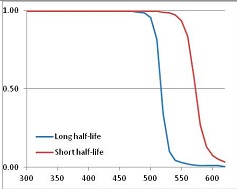
Isoleucine	A	[103,105,128]						582.3	620.8
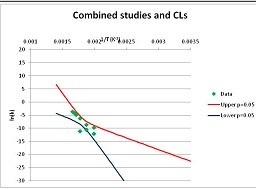
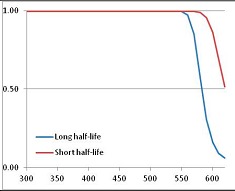
Isomaltose	S	[111]						478.4	509.1
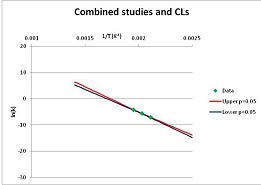
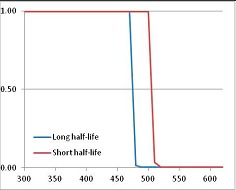
Lactose	S	[111]						450.9	499.4
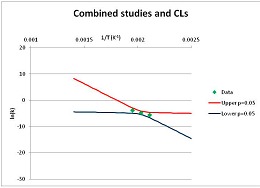
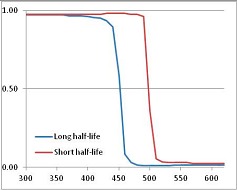
Leucine	A	[103,105,128,129,130]			45	[57,58]		563.8	601.8
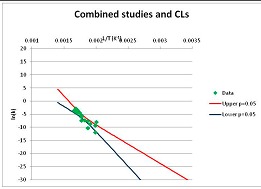
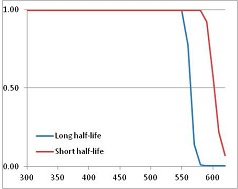
Long chain triglycerides	L	[131]						340.1	397.8
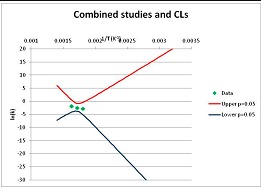
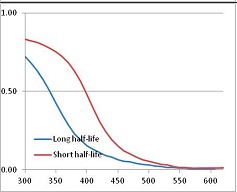
Lyxose	S	[49]						474.9	518.2
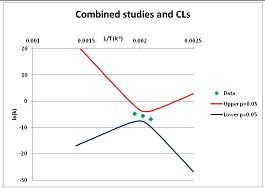
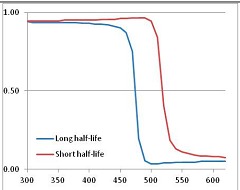
Malonate	I	[132,133]						456.4	515.9
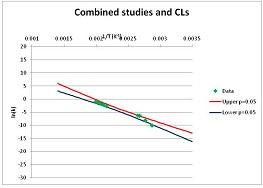
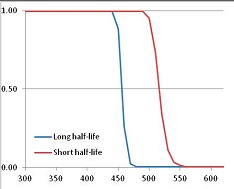
Maltose	S	[47,111]						470.3	509.7
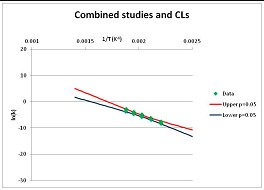
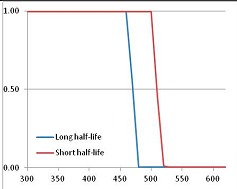
Mandelic Acid (racemization)	I							605	665.2
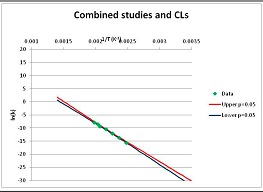
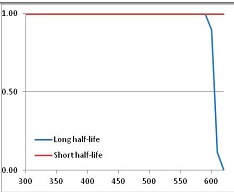
Mannose	S	[47]						463.9	515.7
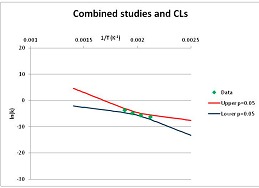
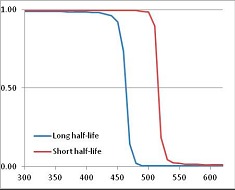
Melibiose	S	[111]						462	494
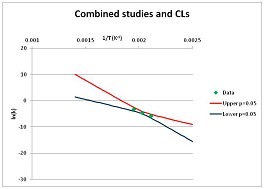
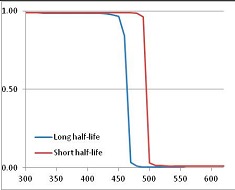
Methionine	A	[103,105,128]						546.5	581.3
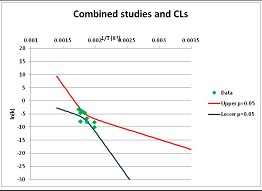
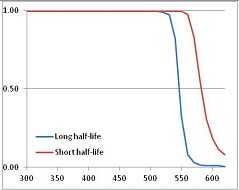
N-acetyl glucosamine	D	[125]						429.5	458.8
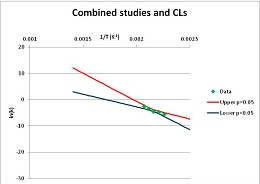
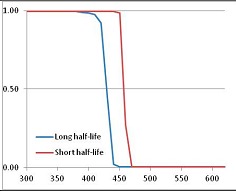
NADH	C	[27,28,29,30,31]						352.3	372.2
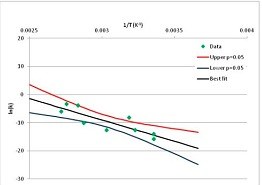
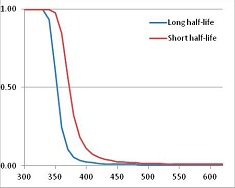
Orotic acid	I	[134]						605	665.2
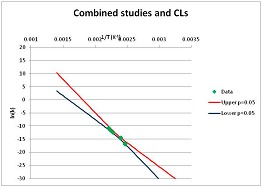
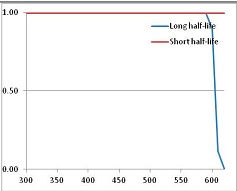
Oxaloacetate	T	[135]			319 1000	2.7		328.5	355.8
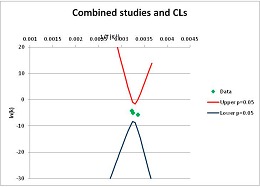
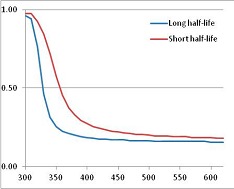
Palatinose	S	[111]						459.1	493.3
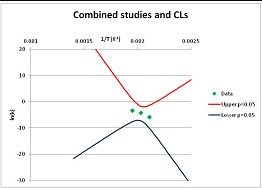
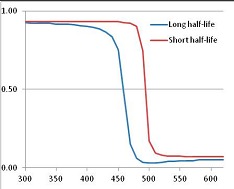
Phenylalanine	A	[103,105,128,129]			180	[59]		569.1	613.6
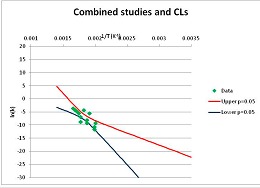
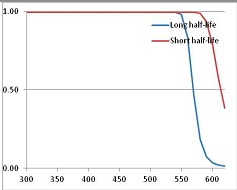
Phosphatidyl choline	L	[136]			3000 (phosphatidyl glycerol) 3600	[61,62]		472.3	544.3
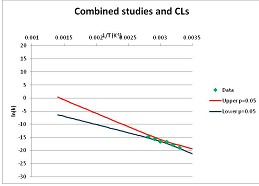
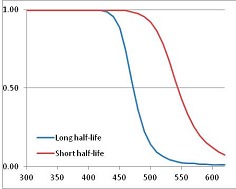
Proline	A	[105]						601.1	640.1
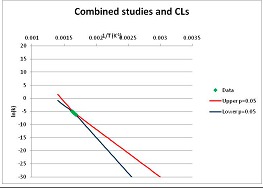
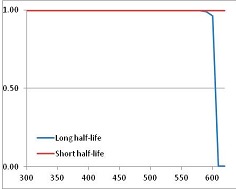
Pyruvate	G	[137]			294	[63]		570.9	620.6
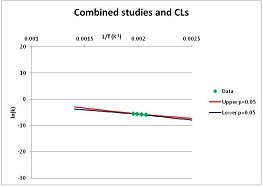
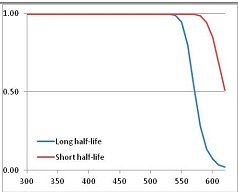
Ribose	S	[49,138]						440.4	516.3
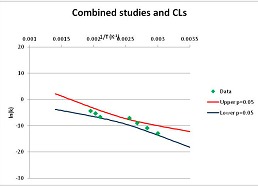
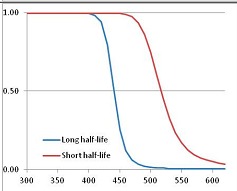
Serine	A	[103,105,106,128]						525.7	560.9
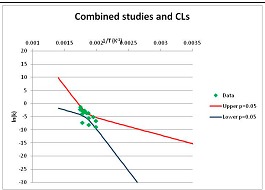
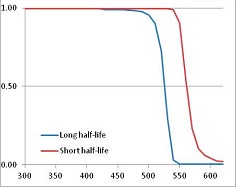
Sucrose	S	[139]						375.5	430.3
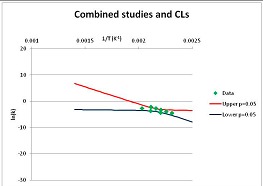
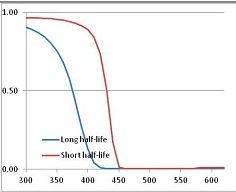
Threonine	A	[105]						516	554.7
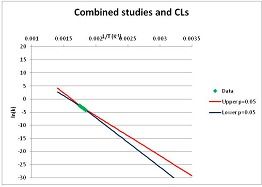
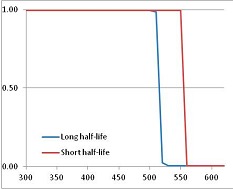
Thymidine	N	[100]						552.4	597.1
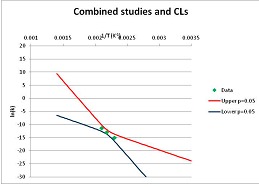
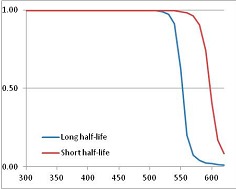
Trehalose	S	[111]						496.1	525.8
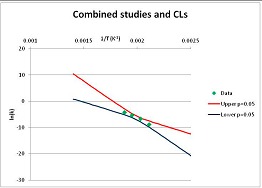
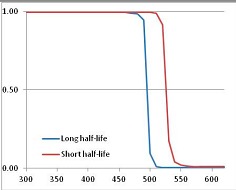
Turanose	S	[111]						449.3	478.1
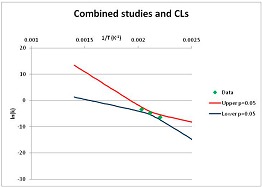
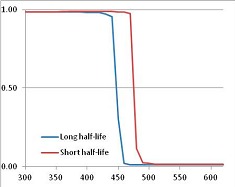
Tyrosine	A	[128]						589.5	710.4
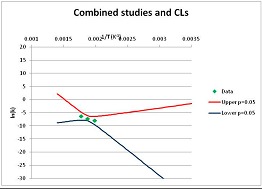
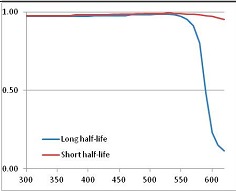
Uracil	N	[100]						552.4	597.1
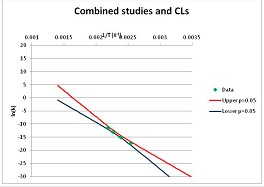
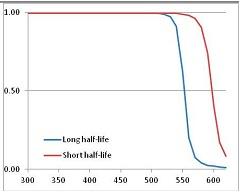
Urea	I	[140]						446.3	475.5
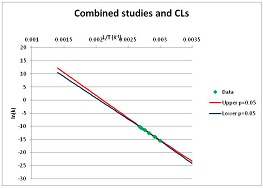
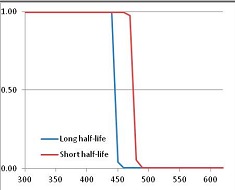
Valine	A	[103,105,130]			40	[56]		577.4	600.6
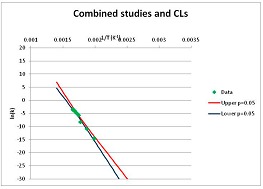
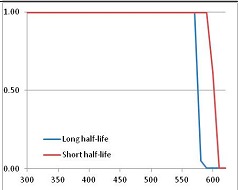
Xanthine	I	[100]						595	660.5
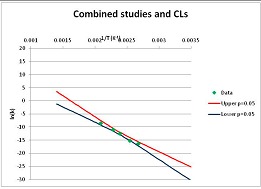
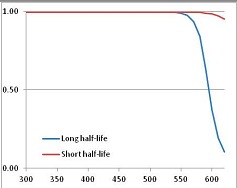
Xylose	S	[48,49,50]						434	482.3
